# Potential for Recycling Metakaolin/Slag-Based Geopolymer Concrete of Various Strength Levels in Freeze–Thaw Conditions

**DOI:** 10.3390/ma17091944

**Published:** 2024-04-23

**Authors:** Mengtong Liu, Hui Liu, Minqi Hua, Chunhong Chen, Xinjie Wang, Xiang Guo, Tianyu Ma

**Affiliations:** 1 School of Urban Construction, Changzhou University, 21 Gehu Middle Road, Wujin District, Changzhou 213164, China; liumengtong97@163.com (M.L.); chench@cczu.edu.cn (C.C.); wangxinjie@cczu.edu.cn (X.W.); guoxiang2131@cczu.edu.cn (X.G.); 20447120@smail.cczu.edu.cn (T.M.); 2 School of Civil Engineering & Architecture, Wuhan University of Technology, Wuhan 430070, China; hmq@whut.edu.cn

**Keywords:** geopolymer concrete, recycling potential, strength levels, freeze–thaw resistance, recycled geopolymer aggregates, physical properties

## Abstract

Geopolymer concrete (GPC) represents an innovative green and low-carbon construction material, offering a viable alternative to ordinary Portland cement concrete (OPC) in building applications. However, existing studies tend to overlook the recyclability aspect of GPC for future use. Various structural applications necessitate the use of concrete with distinct strength characteristics. The recyclability of the parent concrete is influenced by these varying strengths. This study examined the recycling potential of GPC across a spectrum of strength grades (40, 60, 80, and 100 MPa, marked as C40, C60, C80, and C100) when subjected to freeze–thaw conditions. Recycling 5–16 mm recycled geopolymer coarse aggregate (RGAs) from GPC prepared from 5 to 16 mm natural coarse aggregates (NAs). The cementitious material comprised 60% metakaolin and 40% slag, with natural gravel serving as the NAs, and the alkali activator consisting of sodium hydroxide solution and sodium silicate solution. The strength of the GPC was modulated by altering the Na/Al ratio. After 350 freeze–thaw cycles, the GPC specimens underwent crushing, washing, and sieving to produce RGAs. Subsequently, their physical properties (apparent density, water absorption, crushing index, and attached mortar content and microstructure (microhardness, SEM, and XRD) were thoroughly examined. The findings indicated that GPC with strength grades of C100, C80, and C60 were capable of enduring 350 freeze–thaw cycles, in contrast to C40, which did not withstand these conditions. RGAs derived from GPC of strength grades C100 and C80 complied with the criteria for Class II recycled aggregates, whereas RGAs produced from GPC of strength grade C60 aligned with the Class III level. A higher-strength grade in the parent concrete correlated with enhanced performance characteristics in the resulting recycled aggregates.

## 1. Introduction

Elevated carbon emissions contribute to rising global temperatures and intensify the greenhouse effect [1,2]. Cement, as a fundamental consumable in the construction industry, necessitates mining and high-temperature calcination processes. These activities not only cause environmental degradation but also result in substantial CO_2_ emissions [3,4]. Geopolymer, an emerging green building material, is predominantly an inorganic polymer featuring a three-dimensional network structure. It is synthesized through the depolymerization and polycondensation reactions of silicon–aluminum oxides found in materials such as fly ash, metakaolin, and red mud, under the action of activators, including acids, alkalis, and salts [5]. Geopolymer is considered an excellent substitute for traditional ordinary Portland cement (OPC), thanks to its superior qualities, including high strength, robust stability, good acid and corrosion resistance, and fire resistance [6,7,8]. Its manufacturing process emits negligible CO_2_, making it an environmentally friendly option. Producing 1 ton of traditional Portland cement emits approximately 900–1000 kg of CO_2_, while producing 1 ton of geopolymer cement only emits 200–300 kg of CO_2_, which means that using geopolymer instead of traditional Portland cement can reduce carbon emissions by about 70% [9,10].

Cold regions refer to areas where the average temperature of the coldest month meets −10 °C–0 °C every year, and the number of days with a daily average temperature of ≤5 °C is 90–145 days every year [11]. Cold regions are widely distributed across the world, encompassing approximately 25% of the land area [12]. These regions are primarily located in the northern parts of North America, Europe, and Asia, as well as in Central Asia, Antarctica, and its surrounding islands. In China, the cold regions account for about 40% of the country’s total area [13]. Therefore, frost resistance becomes one of the most important durability characteristics for concrete structures in these regions [14]. In such an environment, concrete constructions have a longer setting time, slower strength development, and a reduced dehydration rate [15], as well as water that penetrates the concrete and undergoes freezing and melting to generate expansion pressure, which escalates the quantity of internal cracks and voids within the concrete, thus reducing its durability [16,17]. With the influence of recurrent freeze–thaw cycles, the structure of buildings in cold regions transitions from compact to porous, markedly reducing the buildings’ service life. Research on the frost resistance of OPC concrete indicates that incorporating air-entraining agents (AEAs) and fibers can markedly boost its durability against freeze–thaw cycles [18,19,20]. AEAs work by creating micro air bubbles of 20 to 50 µm, offering space for water to freeze and expand, which in turn lowers the internal pressure of the concrete [21,22]. Meanwhile, fibers serve to prevent the spread of cracks and reduce stress concentration at the tips of cracks [23,24,25]. However, it has been established in existing research that AEAs and fibers provide limited benefits in enhancing the frost resistance of geopolymer concrete (GPC) [26,27,28]. Pilehvar et al. [29] prepared GPC with a strength of around 80 MPa, using fly ash and slag, and found that, after 28 freeze–thaw cycles, its compressive strength loss was within 5%. Aiken et al. [30] prepared fly ash-based GPC with a strength range of 15–60 MPa and found that, as the slag content increased, the compressive strength and frost resistance of GPC gradually improved. After 56 freeze–thaw cycles, the mass loss of GPC with slag content of 40% and 70% was only 2.6% and 0.2%, respectively. Fu et al. [31] used alkaline activator to prepare slag-based GPC with a strength of about 90 MPa. It was found that, after 300 freeze–thaw cycles, its RDEM reached 90%, and the mass loss was only 0.12%. Consequently, to explore the applicability of GPC in cold regions, this study utilized slag as an admixture to formulate a metakaolin/slag-based binary system geopolymer concrete.

When building structures reach the end of their service life or need to be demolished for reconstruction, many construction and demolition wastes (C&DWs) are generated. Converting this solid waste into recycled aggregates for structural use is a preferred solution to address the waste issue. Figiela et al. [32] used clay bricks and concrete fragments to partially replace metakaolin and fly ash in the production of geopolymers, resulting in geopolymers with better mechanical properties. Korniejenko [33] used mining waste coal shale as a precursor to prepare geopolymer and found that its compressive and bending strength met the requirements of buildings, such as foundations, walls, and columns. Alhawat et al. [34] found that geopolymers have broad application prospects in the construction field, and it is possible to produce GPC with properties comparable to OPC using C&DW-based materials. Current practices for C&DWs’ disposal fail to consider the influence of the parent concrete’s performance on the quality of subsequent recycled aggregates. This oversight results in variability and inconsistency in the quality of the recycled materials, which hinders their potential for effective reuse. By assessing the recyclability of concrete at the design stage, materials of varying quality can be promptly recycled and precisely utilized after the end of a building’s lifespan, greatly reducing resource waste and advancing sustainable development. Building on prior research, GPC is believed to possess promising recycling potential. Akbarnezhad et al. [35] observed that recycled geopolymer aggregates (RGAs) exhibited greater wear resistance than traditional recycled aggregates. Zhu et al. [36] reported that the properties of geopolymer mortar made from recycled geopolymer fine aggregates with a replacement ratio of less than 50% were comparable to those made with natural fine aggregates. Additionally, Mesgari et al. [37] discovered that recycled geopolymer aggregates (RGAs) demonstrated superior bonding performance with geopolymer mortar compared to recycled cement concrete aggregates. Hence, exploring the influence of parent GPC on the properties of RGAs and contemplating the sustainable reuse of waste concrete from the design phase are of paramount importance.

Metakaolin, produced through the low-temperature calcination of ultrafine kaolin, is an amorphous aluminum silicate that is known for its significant pozzolanic activity. This makes it a frequently utilized precursor in the manufacture of geopolymers [38]. Granulated blast-furnace slag is a by-product of the blast furnace ironmaking process; it is often used as a mineral admixture due to its high activity [39]. In this study, metakaolin combined with 40% slag and crushed stone aggregates was used to prepare GPC. Considering that different building structures in practice require GPC of varying strength levels, and the strength of the parent GPC directly impacts the performance of the resulting RGAs, it is inadequate to examine only a single strength grade of GPC. Therefore, GPC with strengths of 40, 60, 80, and 100 MPa was formulated to evaluate its frost resistance and the recycling potential after reaching a designed service life of 50 years. The aim of this study was to explore the frost resistance of GPC with different strength levels, analyze the performance differences of RGAs, reveal their differential mechanisms, and evaluate their recycling potential. The novelty of this study is related to the recycling potential of GPC with different strength levels, as it has not been studied before.

## 2. Materials and Methods

### 2.1. Materials and Reagents

#### 2.1.1. Metakaolin and Slag

The metakaolin (MK) was sourced from Gongyi Oushang Refractory Materials Co., Ltd. (Gongyi, China), and the slag (SL) came from Longze Water Purification Materials Co., Ltd. (Zhengzhou, China). It is S95-grade slag and was obtained by granulating the molten material from blast furnace smelting through water quenching and cooling. The chemical compositions of both metakaolin and slag are shown in Table 1. It can be seen from Table 1 that the metakaolin contains 48.88% SiO_2_ and 43.39% Al_2_O_3_. This large amount of silicon aluminum oxide is beneficial to the formation of silicon oxygen tetrahedron and aluminum oxygen tetrahedron in the geopolymer gel [40]. The slag contains 40.57% CaO, which helps to consume water in the geopolymer system, making the geopolymer structure more compact and conducive to strength and frost resistance [41].

#### 2.1.2. Alkali Activator

The sodium silicate (Na_2_SiO_3_) solution, featuring a modulus of 2.31 and a mass fraction of 42%, was purchased from Wuxi City Yatai United Chemical Co., Ltd. (Wuxi, China). The solid sodium hydroxide (NaOH) was obtained from Kunshan Jincheng Reagent Co., Ltd. (Kunshan, China). Its solution with a concentration of 12 mol/L was prepared using distilled water. It was allowed to stand for 24 h prior to usage.

#### 2.1.3. Aggregate

**Figure 1:**
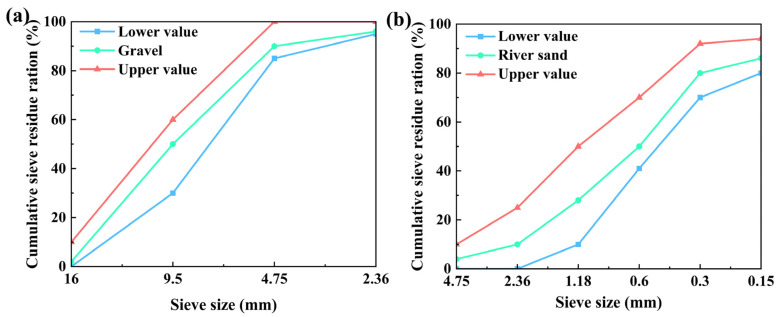
The aggregate gradation: (**a**) coarse aggregate and (**b**) fine aggregate.

The coarse aggregate utilized in this study was natural crushed stone, characterized by a particle size of 5–16 mm and an apparent density of 2738 kg/m^3^. For the fine aggregate, river sand was used, which has a fineness modulus of 2.8 and an apparent density of 2606 kg/m^3^. The aggregate gradations are shown in Figure 1. These aggregates complied with the continuous gradation requirements specified by standards GB/T 14684-2022 [42] and GB/T 14685-2022 [43] (relevant international standard refers to ASTM C33/C33M-23 [44]).

### 2.2. Methods

#### 2.2.1. Preparation of GPC and RGA

Four groups of GPCs with varying strength grades (40, 60, 80, and 100 MPa) were developed, designated as GPC-C40, GPC-C60, GPC-C80, and GPC-C100, respectively.

Referring to previous studies to design the mix ratio [45,46,47,48], a consistent mass of 450 g for the cementitious material was maintained, and the mass ratio of NaOH and Na_2_SiO_3_ solution was fixed at 1:1.6. The strength of GPC was modulated by varying the Na/Al ratio, with pre-experiment conducted for adjustment and optimization. The determined mix proportions are presented in Table 2.

The preparation process of GPC was executed according to the following sequence of steps: Initially, the cementitious material and aggregate were mixed and stirred for approximately 90 s. Subsequently, the NaOH solution, Na_2_SiO_3_ solution, and water were added. After thorough stirring for about 120 s, the mixture was transferred into the mold. Once the surface had sufficiently hardened, the mold was wrapped in plastic and placed in a standard curing room, where it was maintained at a constant temperature of 20 ± 2 °C and a relative humidity exceeding 95%. Twenty-four hours post-casting, the specimens were demolded, sealed in air-tight bags, and placed in an environment maintained at 80 °C for an additional 24 h. After this high-temperature curing phase, the specimens were relocated to the standard curing room to continue the curing process until 3 d, 7 d, 14 d, and 28 d. Cubic specimens with dimensions of 100 mm × 100 mm × 100 mm were employed for assessing mechanical properties, while prismatic specimens measuring 100 mm × 100 mm × 400 mm were used for the frost-resistance test.

Post-freeze–thaw cycling, the GPC specimens were subjected to crushing with a jaw crusher to yield recycled geopolymer aggregates (RGAs). These were correspondingly labeled as RGA-C40, RGA-C60, RGA-C80, and RGA-C100, in alignment with their originating GPC-strength grades.

#### 2.2.2. Test Methods

The compressive strength (f_c_) and splitting tensile (f_t_) strength of GPC at 3 d, 7 d, 14 d, and 28 d was tested in accordance with the specified standard GB/T 50081-2019 [49] (relevant international standards refer to ASTM C39/C39M-23 [50] and ASTM C496/C496M-17 [51]). A microcomputer-controlled pressure testing machine (c) manufactured by Wuxi Xinluda Instrument Equipment Co., Ltd. (Wuxi, China) was used to test the f_c_ and f_t_ of the specimens, with the average value calculated from three specimens in each group.

In accordance with the rapid freeze–thaw cycle method outlined in standard GB/T 50082-2009 [52] (relevant international standard refers to ASTM C666/C666M-15 [53]), the frost resistance of GPC was evaluated based on the relative dynamic modulus of elasticity (RDEM) and mass loss. The average of three samples in each group was taken as the final result. A rapid freeze–thaw cycle testing machine (TDR-3F48) manufactured by Shanghai Sanhao Refrigeration Equipment Factory (Shanghai, China) was employed to assess frost resistance. The test was concluded once the number of freeze–thaw cycles reached 350, the mass loss attained 5%, or when the RDEM fell below 60%.

The apparent density, water absorption, and crushing index of the RGAs were assessed in accordance with the guidelines set forth in GB/T 14685-2022 [43] (relevant international standards refer to ASTM C127-15 [54] and EN 1097-2: 2020 [55]). Thermal treatment methods were used to determine the attached mortar content of RGAs [56]. The average of three samples in each group was taken as the final result. Referring to GB/T 25177-2010 [57], RGAs are classified according to Table 3.

SEM (Zeiss SUPRA55) manufactured by Carl Zeiss AG (Oberkochen, Germany) was employed to examine the microstructural morphology of GPC and RGAs. An electric grinder was used to select samples with dimensions of 5 mm × 5 mm × 3 mm from GPC and RGA, which contained mortar and aggregate. The bottom surface was ground flat, and then it was baked in a 60 °C oven for 24 h. Before testing, the samples were sprayed with gold. The microhardnesses of the RGA’s interfacial transition zone (ITZ) and attached mortar were measured using a digital Vickers microhardness tester (HVS-1000SS) manufactured by Shanghai Precision Instrument Co., Ltd. (Shanghai, China), with a load of 10 g and a loading time of 10 s. XRD (D/Max2500) manufactured by Rigaku (Tokyo, Japan) was utilized to characterize the mineral compositions of the attached mortar on the RGA. After extracting the attached mortar, a mortar was used to grind it into a flour shape, and then it was baked in a 60 °C oven for 24 h.

## 3. Results and Discussion

### 3.1. Compressive Strength of GPC

**Figure 2:**
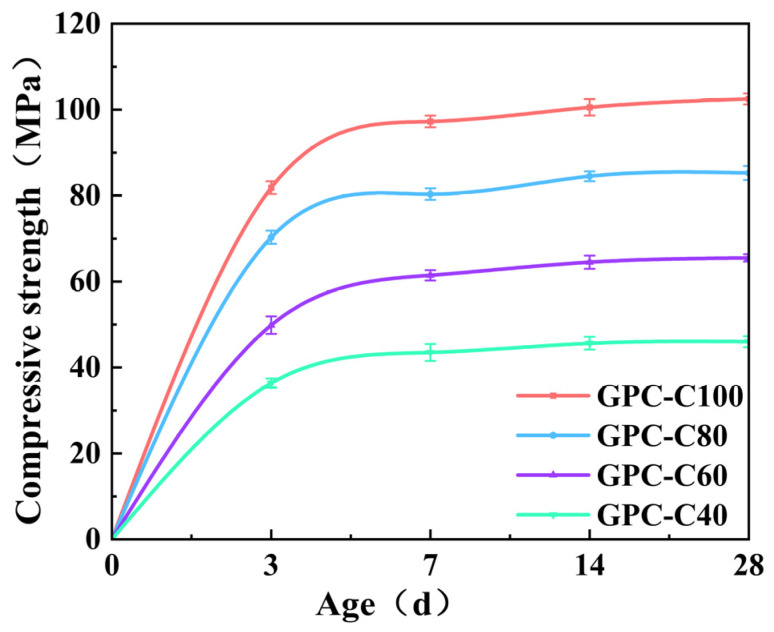
Compressive strength development of GPC specimens.

The compressive strength evolution of the four GPC specimen groups at 3 d, 7 d, 14 d, and 28 d, before undergoing the freeze–thaw cycles, is depicted in Figure 2. Regardless of the strength grade, all the compressive strengths of the GPC showed a rapid increase within the initial seven days, subsequently experiencing a gradual slowdown. By the 7th day, the specimens across all four groups had essentially achieved their anticipated strength levels. The 7-day compressive strengths of GPC-C100, GPC-C80, GPC-C60, and GPC-C40 attained 94.9%, 94.3%, 93.8%, and 94.6% of their respective 28-day compressive strengths, which was attributed to the application of high-temperature curing. By curing at 80 °C for a single day, the efficiency of the polymerization reaction was markedly improved, which shortened the overall curing duration for GPC and significantly enhanced its early strength development [58,59]. Beyond the initial 7 d, the increase rate of compressive strength in GPC began to taper off.

Early in the curing process, the presence of high levels of active ingredients in the cementitious materials, combined with alkali activators, fosters swift dissolution of silico-aluminate components [60]. This action swiftly triggers hydration and geopolymerization reactions, leading to the formation of polymer gels, which fill the pores within the material, significantly enhancing its density and strength [61]. Consequently, this sequence of events is instrumental in contributing to the rapid escalation of early strength. But over time, these reactions slow down as the material approaches its final structure and properties. With the decrease in alkali dosage, the final strength of GPC decreases, indicating that the dosage of the alkali activator will affect the strength of GPC. The higher the amount of alkali activator, the faster the silicon–aluminum oxide in the cementitious material will be dissolved, and the more complete the polymerization reaction will be [62], resulting in the formation of more geopolymer gels and higher strength.

### 3.2. Splitting Tensile Strength of GPC

**Figure 3:**
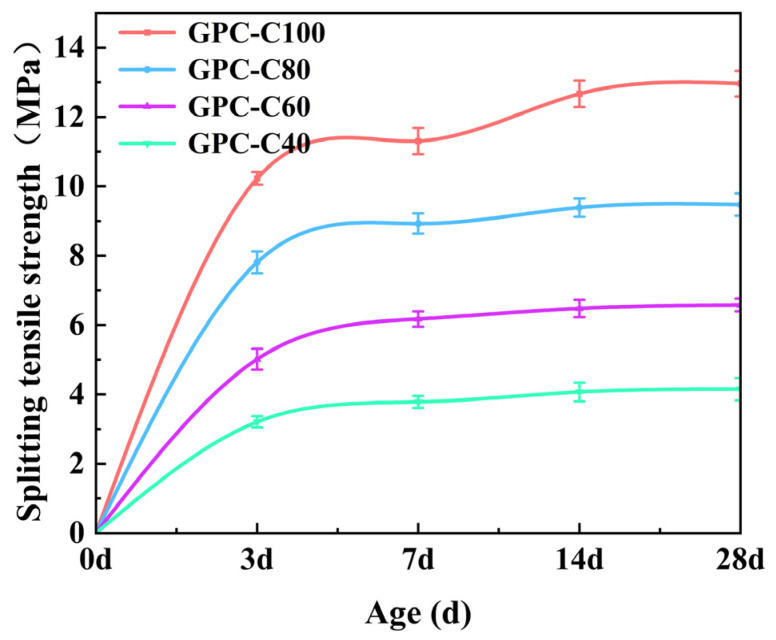
Splitting tensile strength development of GPC specimens.

The splitting tensile strength of the samples before freeze–thaw cycles at 3 d, 7 d, 14 d, and 28 d is shown in Figure 3. As the age increases, the splitting tensile strength increases rapidly in the initial stage and gradually slows down in the later stage. The 7d splitting tensile strength of GPC-C100, GPC-C80, GPC-C60, and GPC-C40 reached 87.2%, 94.3%, 93.8%, and 91.1% of their 28-day splitting tensile strength, respectively. The splitting tensile strength and compressive strength are positively correlated. GPC-C100 has the highest 28-day splitting tensile strength, at 12.96 MPa, while GPC-C40 has the lowest 28-day splitting tensile strength, at 4.15 MPa. The content of calcium oxide and the amount of alkali activator have an important impact on the strength of GPC. Within a certain range, the greater the content of calcium oxide is and the greater the amount of alkali activator is, the more conducive to the polymerization reaction [63,64]. Increasing the amount of alkali activator will accelerate the dissolution rate of aluminosilicate, promote the fixation of water and the formation of the structure, accelerate the rapid setting and formation of geopolymer gel, and produce a compact and solid structure, which is conducive to the development of early strength. At the same time, high-temperature curing will also accelerate this process [65].

### 3.3. Frost Resistance of GPC

#### 3.3.1. Visual Appearance

**Figure 4:**
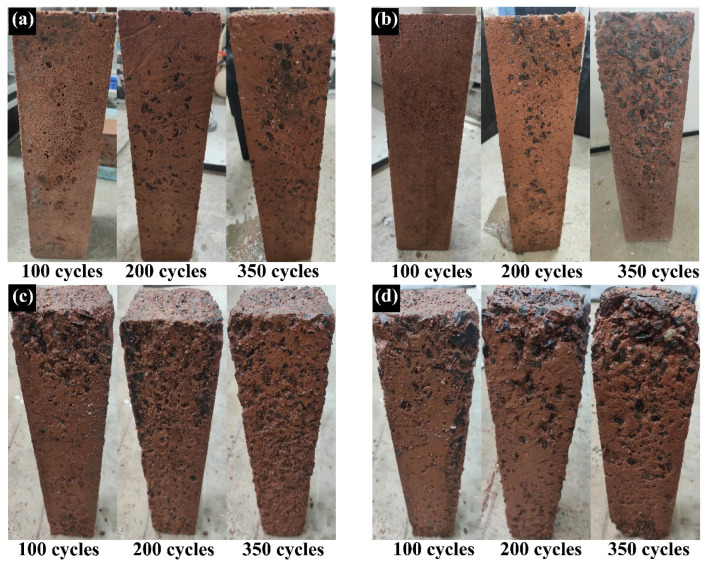
Visual appearance of GPC specimens: (**a**) GPC-C100, (**b**) GPC-C80, (**c**) GPC-C60, and (**d**) GPC-C40.

Figure 4 illustrates the degradation process of the four GPC specimen groups when exposed to freeze–thaw cycles. The figure reveals a trend where the damage to the GPC specimens intensifies with diminishing strength grades. By the 100th cycle, the surface geopolymer mortar of GPC-C100 and GPC-C80 showed minor peeling, while GPC-C60 and GPC-C40 experienced more pronounced peeling. Upon reaching the 200th cycle, a significant portion of the surface mortar on GPC-C100 and GPC-C80 had peeled away, revealing small areas of aggregate, whereas most of the aggregate in GPC-C60 and GPC-C40 had become exposed. After 350 cycles, all specimens except for GPC-100 showed severe peeling of the geopolymer mortar. As depicted in the figure, the freeze–thaw damage process for GPC is characterized by continual surface peeling, with an initial tendency for slow damage progression that later accelerates. As shown in the figure, the freeze–thaw damage process of GPC is characterized by continuous surface peeling. Initially, the damage progressed slowly, but it later accelerated, and the damage at both ends was more severe than in the middle. It is worth noting that no cracks were observed in all samples during the freeze–thaw stage.

The findings indicated that higher strength correlated with a denser concrete structure, making it more challenging for water to infiltrate. Furthermore, a stronger bond between the aggregate and mortar enhanced the concrete’s capacity to withstand the expansive pressure exerted by freezing water, thereby reducing the likelihood of crack and pore formation.

#### 3.3.2. RDEM and Mass Loss

**Figure 5:**
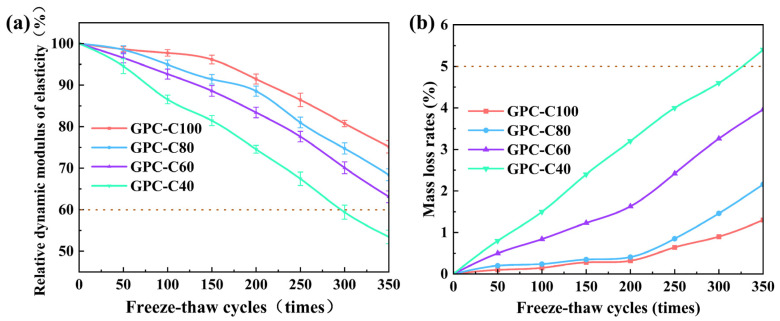
Relative dynamic modulus of elasticity (RDEM) and mass loss rates of GPC specimens: (**a**) RDEM, (**b**) mass loss rates.

The RDEM and mass loss of the four groups of specimens after the freeze–thaw cycles are shown in Figure 5. The trend of RDEM and mass loss is consistent with the freeze–thaw damage process. As the number of freeze–thaw cycles increased, the RDEM and mass loss for GPC-C40 exhibited a swift decline. After 350 cycles, the RDEM dipped below 60%, and the mass loss exceeded 5%, signifying that GPC-C40 fell short of the durability criteria necessary for a projected 50-year lifespan in cold climates. The subpar performance of GPC-C40 was primarily due to an insufficient polymerization reaction, which was a consequence of the minimal alkali activator present, resulting in a lower quantity of geopolymer gel. The unreacted metakaolin in the composition led to increased porosity, which allowed for quicker water absorption. Consequently, once cracks began to form, they rapidly widened, interconnected, and progressed into larger and more severe fissures. Therefore, an appropriate amount of alkaline activator has an important impact on the performance of GPC.

The GPC-C100, GPC-C80, and GPC-C60 exhibited minimal changes in RDEM and mass loss at the 200-cycle mark. Specifically, the RDEM values were 91.5%, 88.6%, and 83.4%, respectively, while the corresponding mass loss rates were 0.32%, 0.41%, and 1.63%. Following 200 cycles, the RDEM and mass loss for the specimens began to decline swiftly. By the 350th cycle, the RDEM figures had fallen to 75.2%, 68.4%, and 63.1%, respectively, and mass loss had increased to 1.3%, 2.16%, and 3.96%, respectively. This trend could be attributed to the initially dense structure and high strength of the concrete, which impeded moisture penetration, resulting in a gradual change in RDEM and mass loss in the early stages. However, as the freeze–thaw process progressed and the surface mortar of the GPC started to peel off, the strength diminished, and moisture ingress became more pronounced. This led to the formation of larger pores and wider cracks within the GPC, thereby accelerating the decline in RDEM and the increase in mass loss.

As illustrated in the Figure 5, the greater the GPC’s ability to resist the expansion of pores and cracks, the slower the rate of decline in RDEM and mass loss. GPC-C100, GPC-C80, and GPC-C60 all demonstrated the potential to withstand 50 years of service in the cold-climate regions.

#### 3.3.3. SEM Analysis

**Figure 6:**
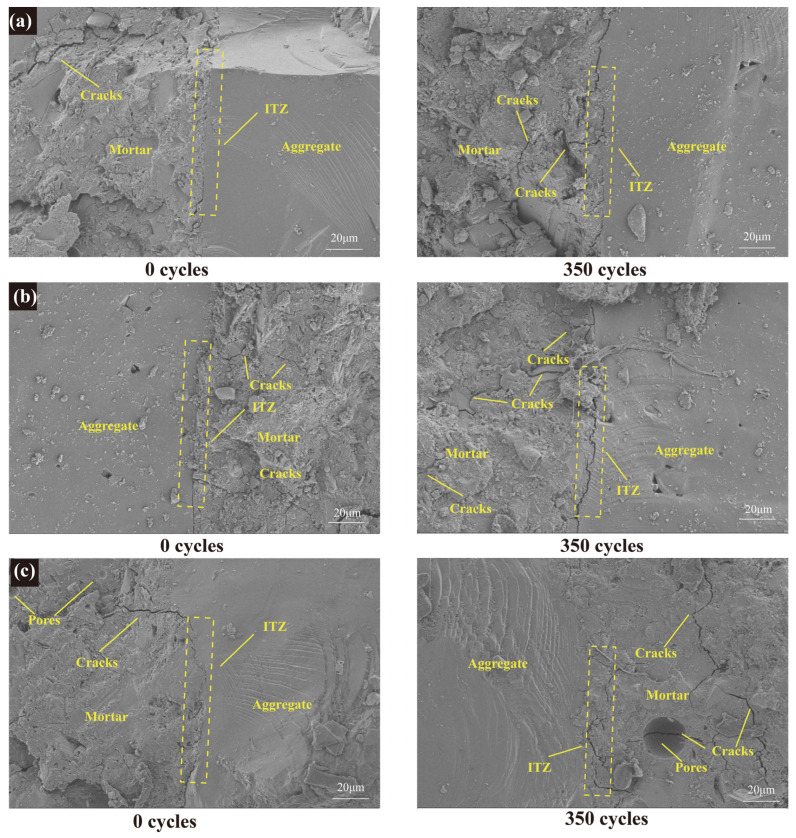
SEM images of GPC before and after freeze–thaw cycles: (**a**) GPC-C100, (**b**) GPC-C80, and (**c**) GPC-C60.

The SEM images of GPC with different strength levels before and after freeze–thaw cycles are shown in Figure 6, indicating that the macroscopic strength differences are reflected at the microscopic level. Before freeze–thaw cycles, the three groups of samples were relatively dense and uniform, and the ITZ between aggregate and mortar was relatively tight, because the addition of slag made the geopolymer react to generate new geopolymer gel, filling the pores. As the number of freeze–thaw cycles increased, the microstructure of the three specimens showed different changes. After 350 freeze–thaw cycles, GPC-C100 continued to maintain its dense mortar structure and ITZ, which slightly widened. Only a small number of fine cracks appeared in the mortar, indicating that the effect of the freeze–thaw cycles had the smallest impact on it. The microstructure images of GPC-C80 and GPC-C60 show that, after 350 freeze–thaw cycles, their ITZ was further widened, resulting in cracks and pores of different sizes and quantities; also, some cracks penetrated the pores, and this may be the reason for the drastic changes in their RDEM. It shows that the strength grade will affect the effect of freezing and thawing. The higher the strength, the stronger the GPC’s ability to resist crack and pore expansion. This is because it has sufficient geopolymer gel, which is tightly combined, thus making it difficult for crack and pore expansion. ITZ with lower strength has more unreacted metakaolin particles, which can increase the porosity of GPC and increase harmful pores, which is unfavorable for frost resistance [66].

### 3.4. Properties of RGA

#### 3.4.1. RGA Gradation

**Figure 7:**
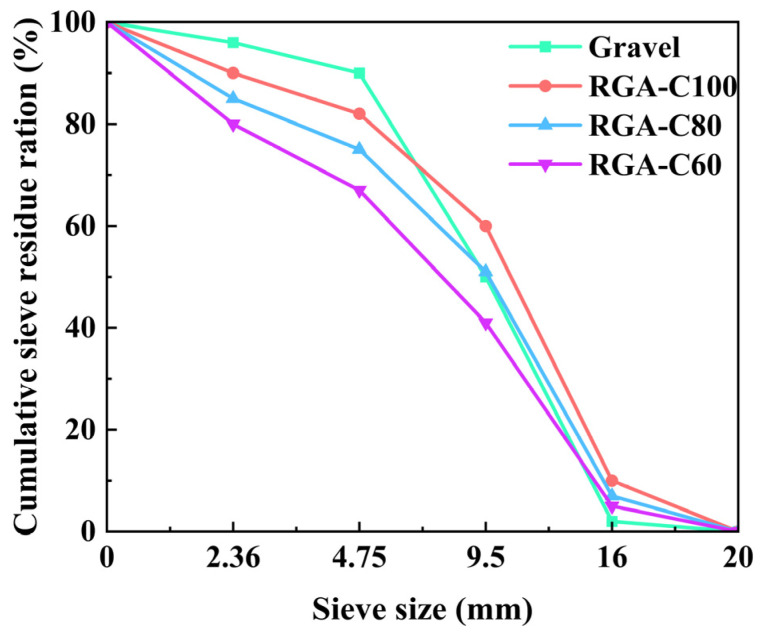
The RGA gradation.

To explore the recycling potential of GPC after enduring freeze–thaw cycles equivalent to a 50-year service life, GPC specimens that survived 350 such cycles were crushed to produce RGA. Given that GPC-C40 exhibited failure following 350 freeze–thaw cycles and considering that failed concrete was not deemed suitable for recycling into aggregates, this concrete was not used to produce RGA-C40 within the scope of the experiment. After undergoing cleaning and screening, the gradations of the other three RGA groups were determined, as depicted in Figure 7. Notably, their gradations significantly diverged from that of natural aggregates (NAs).

The RGA-C100 and RGA-C80 specimens contained a greater proportion of large particle-size aggregates compared to NAs. This was attributed to the stronger bond between the mortar and the aggregate in these parent geopolymer concretes, which inhibited their separation and led to a higher mortar content, thus contributing to an increase in particle size. Conversely, RGA-C60 showcased a higher quantity of fine particle-size aggregates relative to natural aggregates. This was because the bonding force of the attached mortar and the aggregate was weak, which allowed the mortar to detach more easily under the action of extrusion force. Consequently, the strength level of the parent concrete had a significant impact on the particle size distribution of the next generation of recycled aggregates.

#### 3.4.2. Physical Properties of RGA

**Figure 8:**
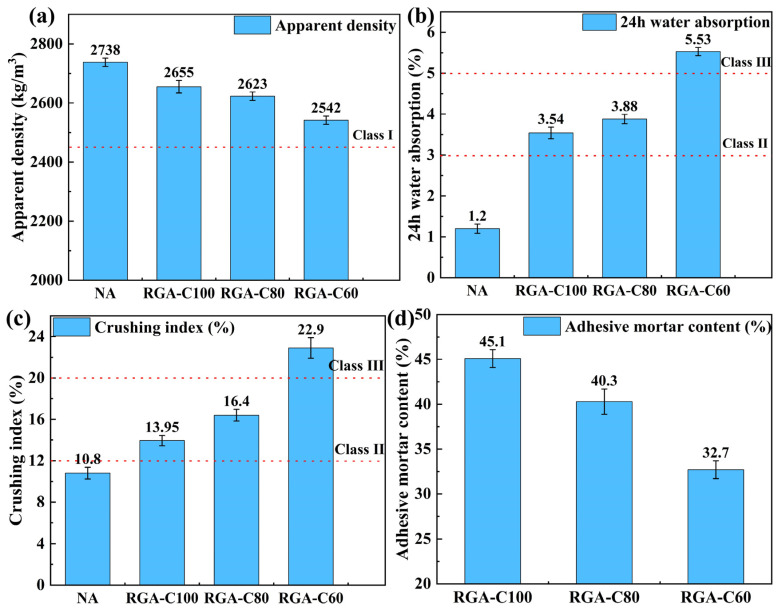
Physical properties of NA, RGA-C100, RGA-C80, and RGA-C60: (**a**) apparent density, (**b**) 24 h water absorption, (**c**) crushing index, (**d**) adhesive mortar content.

Variations in aggregate gradation can result in considerable disparities in its performance. To facilitate a more accurate comparison of their properties, the performance of the RGA was assessed after their gradations were reconfigured to match that of NA through sieving. The physical properties of the RGA are depicted in Figure 8.

Compared with NAs, the apparent density of RGA-C100, RGA-C80, and RGA-C60 diminished by 3.03%, 4.2%, and 7.1%, respectively. Concurrently, the 24-h water absorption rates were elevated by 1.95, 2.23, and 3.61 times, while the crushing indices escalated by 29.2%, 51.9%, and 112%, in that order. Despite these changes, the apparent densities of all three RGAs still meet the criteria for Class I recycled aggregate laid out in GB/T 25177-2010 [57]. The water absorption rate and crushing index for RGA-C100 and RGA-C80 satisfied the criteria for Class II recycled aggregate, whereas RGA-C60 aligned with the Class III recycled aggregate standards.

This demonstrated that all three GPC groups possessed potential for recycling. The freeze–thaw cycles contributed to an increased prevalence of wide cracks and micro-pores in the geopolymer mortar and ITZ, concurrently weakening the bonding force between the mortar and aggregate [67]. Such changes led to a reduction in the performance of RGA. Notably, a higher strength of the parent GPC correlated with greater strength in the mortar and ITZ of the RGA [68], thus enhancing the overall performance of the recycled product.

### 3.5. Microstructure of RGA

#### 3.5.1. Microhardness

**Figure 9:**
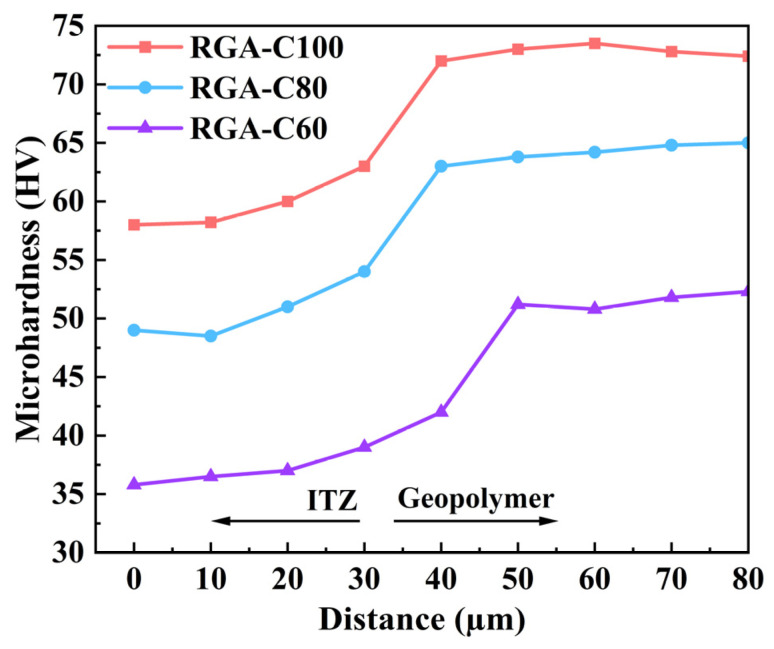
Microhardness distribution of RGA from ITZ to geopolymer mortar.

The hardness values for RGA from ITZ to attached mortar are shown in Figure 9. The microhardness of RGA-C100 and RGA-C80 shows a slow change trend at 0–10 µm, while the microhardness of RGA-C60 shows a slow change at 0–20 µm, indicating that it should be located in the ITZ of RGA, and the slight change may be due to the contact with pores or cracks during the measurement process. RGA-C100 and RGA-C80 showed a significant increase in microhardness at 10–40 µm, while RGA-C60 showed a significant increase in microhardness at 20–50 µm, indicating that this should be located in the junction between ITZ and the geopolymer mortar, which is jointly affected by ITZ and the geopolymer mortar. As the distance increases, the portion borne by the geopolymer mortar increases; therefore, the microhardness values increase. The microhardness tends to flatten at 50–80 µm, indicating that it should be located in the geopolymer mortar. The average microhardness values of the ITZs for RGA-C100, RGA-C80, and RGA-C60 were 58.1, 49.3, and 35.8, while the attached mortar exhibited average values of 72.8, 64.6, and 51.2, respectively. The microhardness of the GPC’s ITZs corresponded to 79.8%, 76.3%, and 68.5% of the attached mortar’s, respectively. This indicated that the higher the strength of the parent GPC, the greater the strength of both the ITZs and attached mortar in the RGA, and the narrower the gap between the strength of the ITZs and the attached mortar.

The higher strength of the parent GPC resulted in a denser and more homogenously distributed structure within the ITZs and attached mortar. Subsequently, this enhanced the bonding force with the aggregate, leading to a closer alignment in strength between the ITZs and the attached mortar [69].

#### 3.5.2. SEM Analysis

**Figure 10:**
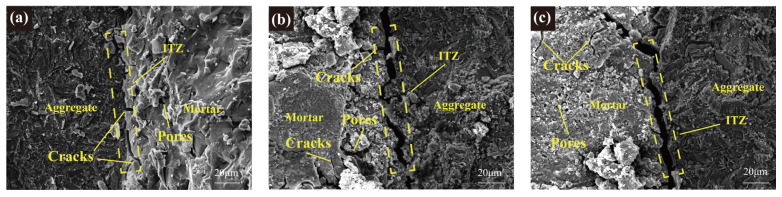
SEM images of RGA: (**a**) RGA-C100, (**b**) RGA-C80, and (**c**) RGA-C60.

Figure 10 displays SEM images of the RGA, revealing a variety of cracks and pores within the attached mortar. Some of these cracks interweaved, creating a networked structure that intersected with the ITZ. This interconnectivity was a contributing factor to the observed reduction in RGA performance. Under the action of freeze–thaw cycles, the water within the pores was repeatedly frozen and thawed, generating cyclic expansion pressure. This process led to the progressive evolution of microcracks and small pores [70], and this further intensified after crushing. Eventually, these microcracks and small pores interconnected and expanded into larger cracks and pores. The physical performance of RGA was inversely proportional to the number of cracks and pores presented. The fewer the defects, the more superior the performance of RGA. RGA-C100 exhibited the narrowest ITZ width, suggesting that its attached mortar was more securely bonded to the aggregate and possessed the most robust resistance to damage. This result was consistent with the macroscopic performance observed.

#### 3.5.3. XRD Analysis

**Figure 11:**
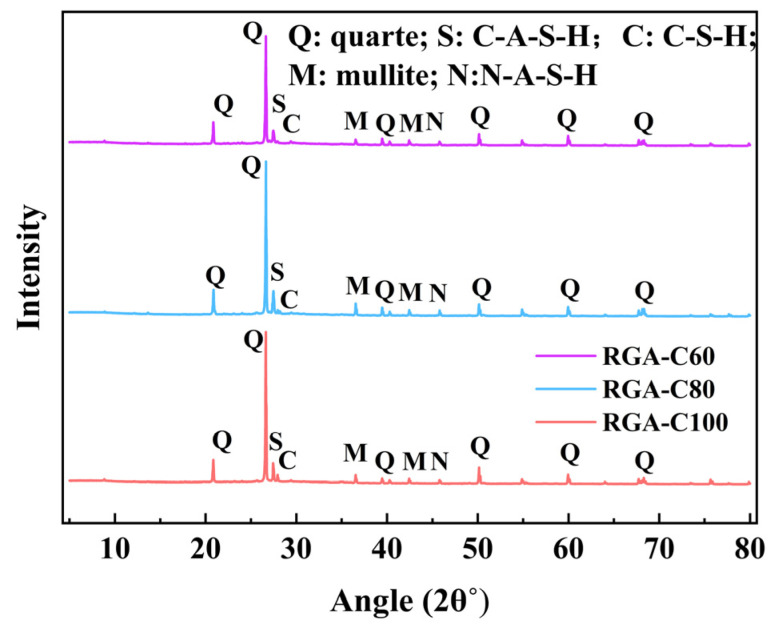
XRD analysis of RGA geopolymer mortar.

Figure 11 presents the XRD analysis of the attached mortar on RGAs, illustrating that, while the diffraction peaks for the three group specimens are similarly positioned, their intensities vary. At 2θ = 27.46°, the characteristic diffraction peak of hydrated calcium silicate aluminate (C-A-S-H) was observed, and at 2θ = 29.42°, the peak corresponding to hydrated calcium silicate (C-S-H) became apparent.

It was indicated that the incorporation of slag results in the formation of higher-strength C-A-S-H and C-S-H. As the compressive strength increases, so does the height of the diffraction peaks, signifying that the sample exhibits high crystallinity and a substantial number of crystalline substances [71]. Calcium components play a pivotal role in the microstructure of geopolymers, and an optimal concentration of these components can modify the cementitious products, facilitating the transformation of N-A-S-H gels into C-A-S-H and C-S-H gels. This conversion can lead to enhancements in both the strength and durability of the geopolymer matrix [72,73].

## 4. Conclusions

Through the examination of the frost resistance and recycling potential of metakaolin-based GPC with different strength grades, the following conclusions are drawn:

(1) A high-temperature (80 °C) curing and the addition of 40% slag accelerated the polymerization reaction, so that the 7-day compressive strengths of GPC-C100, GPC-C80, GPC-C60, and GPC-C40 attained 94.9%, 94.3%, 93.8%, and 94.6% of their respective 28-day compressive strengths. With the exception of GPC-C100, concretes of lower-strength grades generally achieve their designated strength targets more readily within 7 days. The development trend of splitting tensile strength is similar to compressive strength. The 7-day splitting tensile strength of GPC-C100, GPC-C80, GPC-C60, and GPC-C40 reached 87.2%, 94.3%, 93.8%, and 91.1% of their 28-day splitting tensile strength, respectively.
(2) High-strength GPC exhibited superior frost-resistance durability. GPC-C100, GPC-C80, and GPC-C60 showed the capability to endure more than 350 freeze–thaw cycles, suggesting that their lifespan in cold regions extended beyond 50 years. By the 350th cycle, the RDEM had fallen to 75.2%, 68.4%, and 63.1%, respectively, and mass loss had increased to 1.3%, 2.16%, and 3.96%, respectively. In contrast, GPC-C40 experienced failure after 300 cycles. The SEM image shows that the GPC sample has a dense ITZ.
(3) The apparent densities of RGA-C100, RGA-C80, and RGA-C60 are 2655 kg/m^3^, 2623 kg/m^3^, 2542 kg/m^3^, respectively, with water absorption rates of 3.54%, 3.88%, and 5.53%, and crushing indices of 13.95%, 16.4%, and 22.9%, respectively. The metrics for RGA-C100 and RGA-C80 aligned with Class II recycled aggregate standards, whereas RGA-C60 conformed to Class III criteria. The higher the strength grade, the better the physical properties of the RGAs, indicating a linear correlation between the strength grade of the parent concrete and the performance of the subsequent generation of recycled aggregates. The microhardness of the GPC’s ITZs corresponded to 79.8%, 76.3%, and 68.5% of the attached mortar’s, respectively. This indicated that the higher the strength of the parent GPC, the greater the strength of both the ITZs and attached mortar in the RGA, and the narrower the gap between the strength of the ITZs and the attached mortar.
(4) The attached mortar and ITZ of RGA exhibited numerous microcracks and pores, which accounted for the deterioration of its physical properties. Moreover, a higher-strength grade of RGA corresponded to a narrower ITZ width, suggesting a more robust resistance to separation. The XRD results showed that the polymerization reaction generated C-S-H and C-A-S-H, which is one of the reasons for their great recycling potential.

## Figures and Tables

**Table 1:** Chemical compositions of MK and SL determined by XRF.

wt/%	SiO_2_	Al_2_O_3_	CaO	Fe_2_O_3_	P_2_O_5_	K_2_O	TiO_2_	SO_3_	MgO	Na_2_O
MK	48.88	43.39	0.983	3.77	0.072	0.141	2.452	0.044	-	-
SL	30.54	15.27	40.57	0.26	-	0.416	0.747	2.03	9.01	0.548

**Table 2:** Mix proportions of 1 m^3^ GPC specimen (kg).

Grade	Metakaolin	Slag	Gravel	River Sand	Na_2_SiO_3_ Solution	NaOH Solution	Water
C100	270	180	1210	650	206	129	0
C80	270	180	1210	650	184	115	15
C60	270	180	1210	650	162	101	30
C40	270	180	1210	650	131	81	53

**Table 3:** RGAs’ classification standards.

	Apparent Density (kg/m^3^)	Water Absorption Rate (%)	Crushing Index (%)
Class Ⅰ	>2450	<3	<12
Class Ⅱ	>2350	<5	<20
Class Ⅲ	>2250	<8	<30

## Data Availability

Data are contained within the article.
